# A Nationwide Cross-Sectional Survey of Anesthesiology Fellowship Program Directors: Attitudes on Parental Leave in Residency and Fellowship Training

**DOI:** 10.1089/whr.2021.0130

**Published:** 2022-05-06

**Authors:** Molly B. Kraus, Elizabeth B. Malinzak, Rekha Chandrabose, Amy C.S. Pearson, Cindy Ku, Sarah E. Hartlage, Andrew C. Hanson, Phillip J. Schulte, Emily E. Sharpe

**Affiliations:** ^1^ Department of Anesthesiology and Perioperative Medicine, Mayo Clinic Hospital, Phoenix, Arizona, USA.; ^2^ Department of Anesthesiology, Duke University, Durham, North Carolina, USA.; ^3^ Department of Anesthesiology, University of California San Diego, San Diego, California, USA.; ^4^ Department of Anesthesia, University of Iowa, Iowa City, Iowa, USA.; ^5^ Department of Anesthesiology, Queen's Medical Center, Honolulu, Hawaii, USA.; ^6^ Louisville Metro Department of Public Health and Wellness, Louisville, Kentucky, USA.; ^7^ Division of Clinical Trials and Biostatistics, Mayo Clinic, Rochester, Minnesota, USA.; ^8^ Department of Anesthesiology and Perioperative Medicine, Mayo Clinic, Rochester, Minnesota, USA.

**Keywords:** critical care, education, obstetrics, pediatrics, subspecialties

## Abstract

**Introduction:**

Little is known about the impact of parental leave on anesthesiology fellowship directors' perception of their fellows. In addition, use of parental leave during residency can result in “off-cycle” residents applying for a fellowship. This study sought to clarify fellowship directors' attitudes and beliefs on effects of parental leave on fellows and off-cycle fellowship applicants.

**Methods:**

An online survey was sent to anesthesiology fellowship program directors through e-mail addresses obtained from websites of the Accreditation Council for Graduate Medical Education and specialty societies. Descriptive statistical analysis was used.

**Results:**

In total, 101 fellowship directors (31% response rate) completed the survey. Forty-one (41%) directors had a fellow who took maternity leave in the past 3 years. Among the programs, 49 (49%) have a written policy about maternity leave and 36 (36%) have a written paternity or partner leave policy. Overall, most fellowship directors believed that becoming a parent had no impact on fellow performance and professionalism; more respondents perceived a greater negative impact on scholarly activities, standardized test scores, and procedural volume for female trainees than male trainees. Some fellowship directors (10/94; 11%) reported they do not allow off-cycle residents in their program. Among programs that allow off-cycle residents, more directors perceived it a disadvantage rather than an advantage.

**Conclusions:**

Fellowship directors perceive that anesthesiology residents who finish training outside the typical graduation cycle are at a disadvantage for fellowship training.

## Highlights

- Most directors believed that becoming a parent did not affect performance or skill.
- Directors found greater negative effect for women who had children during fellowship.
- Some fellowships (∼10%) do not allow off-cycle residents in their program.
- Directors perceive off-cycle anesthesiology residents to have a disadvantage.

## Introduction

Medical training typically overlaps with the traditional childbearing years, providing challenges for women physicians. Approximately 50% of medical students and 38% of anesthesiologists are women, necessitating close examination of parental leave policies in the training of physicians.^1^ The Family and Medical Leave Act allows up to 12 weeks of parental leave time with job security but does not require pay; however, most physicians in training take considerably less than that.^2^

Residents are often required to prolong their residency training if they use parental leave, resulting in delayed graduation, delayed credentialing board examination eligibility, and “off-cycle” fellowship start dates.^3^ Furthermore, policies made by credentialing boards do not always result in changes to specific residency and fellowship program policy.

In the United States, the American Board of Anesthesiology (ABA) requires diplomats to complete a 12-month internship followed by 36 months of clinical anesthesiology training along with the successful completion of two written board certification examinations, an oral examination, and an Objective Structured Clinical Examination. Fellowship training in anesthesiology is typically 12 months in duration. There are many options for subspecialty fellowship training, including but not limited to the Accreditation Council for Graduate Medical Education (ACGME)-accredited programs of adult cardiothoracic, anesthesiology critical care medicine, obstetric anesthesiology, pain medicine, pediatric anesthesiology, and regional anesthesiology and acute pain medicine.

In 2019, the ABA released its Revised Absence From Training Policy.^4^ This revision allowed the ABA to consider requests for up to 40 additional days away from residency training, reflecting the need for support of trainees for life events (including, but not limited to, parental leave).

Before 2019, trainees were allowed 60 days of absence during the 36-month-long clinical training period and an absence beyond these 60 days (such as parental leave or leave due to illness) required the trainees to extend their training period. In addition, effective from July 1, 2021, the American Board of Medical Specialties released a policy that member boards “must incorporate time away from training for purposes of parental, caregiver, and medical leave in addition to allowed time away for vacation.”^5^ Yet, surveys of residency program directors have revealed lingering concerns and consequences about the impact of a leave on medical training.^6^

We previously published the results of a survey of anesthesiology residency program directors and perceptions of residents who have children during training.^7^ This study aims to examine the beliefs of anesthesiology fellowship directors on the effects of pregnancy and parental leave on fellowship training and their attitude toward off-cycle residents who have taken parental leave during anesthesiology residency and subsequently apply for fellowship.

## Methods

We performed a cross-sectional online survey of anesthesiology fellowship directors' perceptions of parental leave for residents and fellows. The Mayo Clinic Institutional Review Board deemed the present study exempt. An online 45-question survey was adapted from a survey of anesthesiology residency program directors to address anesthesiology fellowship directors, and questions regarding perceptions of off-cycle applicants were added.

The survey included questions about demographic characteristics, program characteristics, program policy about parental leave, coverage of call, and the effects of parental leave on fellow performance (Supplementary Data). The survey was created using Qualtrics software (Qualtrics XM, Provo, UT). After adaptation of the survey, it was piloted with academic faculty at the authors' institutions.

A link to the survey was sent to e-mail addresses for fellowship directors, gathered from the ACGME website for the following subspecialties: obstetrics, pediatrics, chronic pain, and regional anesthesiology and the websites of specialty societies including the Society of Critical Care Anesthesiologists (critical care) and the Society of Cardiovascular Anesthesiologists (cardiac anesthesia). Attempts were made to include other fellowships, such as neuroanesthesia, ambulatory anesthesia, practice management, public policy, transplant anesthesia, and trauma anesthesia. However, obtaining and validating e-mail addresses were difficult, and these fellowships were not included in the analysis.

E-mails were sent directly from Qualtrics in January 2019. Efforts were made to also reach fellowship program directors through listservs, and e-mails were sent through the Society of Pediatric Anesthesiology and the Society of Obstetric Anesthesia and Perinatology. Internet protocol addresses were obtained to ensure no duplicates. Results were anonymous. Data were collected by Qualtrics. Quantitative data were analyzed with the use of Qualtrics software.

Many answers were formatted as a 7-choice Likert-type model with the answer choices *strongly disagree*, *disagree*, *somewhat disagree*, *neither agree nor disagree*, *somewhat agree*, *agree*, and *strongly agree*. In the survey, the term *maternity leave* was defined as childbearing parental leave; *paternity* or *partner leave* was defined as nonchildbearing or adoptive parental leave. An off-cycle resident was defined as a person having an expected completion of residency different than the peer group (after June 30). Program directors were asked whether they allowed off-cycle residents. For responses answered yes, they were asked further questions about the advantages and disadvantages of an applicant being off-cycle.

### Statistical methods

Survey responses were summarized overall, according to the sex of the program director, and according to the program type when provided. Categorical, ordinal, and Likert-type scale survey responses were summarized as number (percentage); continuous survey responses as median (25th percentile and 75th percentile). Survey responses were compared between male and female program directors and among program types using chi-squared tests for questions with yes/no responses and Wilcoxon rank-sum tests (between sexes) or Kruskal–Wallis rank sum tests (among program types) for questions with ordinal or Likert-type scale responses. Finite population correction (FPC) was applied to account for the high proportion of observed program directors in the sample.

When directors were asked about the impact of becoming a parent on various aspects of work, the responses regarding female and male trainees were paired, and Wilcoxon signed-rank tests with FPC were used to assess whether there was a difference in perception of impact on male versus female trainees. The results of the impact questions were presented graphically and compared using the full 7-level scale of responses, but were collapsed into groups of negative, positive, and no impact for tabular presentation.

A complete case analysis was used when complete data were not available. *p* < 0.05 was considered statistically significant. All statistical analyses were performed with R software version 3.6.2 (R Foundation for Statistical Computing, Vienna, Austria).

## Results

In total, 101 anesthesiology fellowship directors completed the survey, or 31% of the 327 fellowship program directors who received the survey. By subspecialty type, the response rates were as follows: cardiac anesthesia 17% (11/66), critical care 33% (18/55), obstetric anesthesia 62% (23/37), pain 15% (13/88), pediatric anesthesia 48% (29/60), and regional anesthesia 29% (6/21).

More than half of the fellowship program directors (58%) reported that women composed 40% or more of their fellows. The majority (58%) of fellowship directors reported that more than 20% of their fellows were parents. Among all directors, 41% had a fellow take maternity leave in the past 3 years. Of the responding program directors, 74% reported maternity leaves of 5 weeks or longer. Twenty-six percent of programs (*N* = 26) had a fellow take paternity leave in the prior 3 years (Table 1).

**Table 1.** General Fellowship Program Information

Survey question^a^	Overall response^b^ (***N*** = 101)
What type of facility is your fellowship program a part of? (*n* = 100)	
Academic	96 (96)
Community	1 (1)
Military	2 (2)
Other, please specify	1 (1)
What type of anesthesiology fellowship do you direct? (*n* = 100)	
Cardiac	11 (11)
Critical care	18 (18)
OB	23 (23)
Pain	13 (13)
Pediatric	29 (29)
Regional and acute pain	6 (6)
Region of the United States (*n* = 98)	
East	25 (26)
Midwest	28 (29)
West	14 (14)
Southeast	12 (12)
South	19 (19)
How many total fellows do you have in your program?	
Median (25th percentile, 75th percentile)	2 (2, 5)
Percentage of female fellows in your program (as a percentage of total fellows in the fellowship program) (*n* = 98)	
0–20	28 (29)
21–40	13 (13)
41–60	29 (30)
61–80	9 (9)
81–100	19 (19)
Percentage of fellows (men and women) with children (*n* = 98)	
0–20	41 (42)
21–40	11 (11)
41–60	20 (20)
61–80	13 (13)
81–100	13 (13)
To your knowledge, does your fellowship program have a written policy (separate from the ABA's or federal/state law) regarding childbearing parental leave, commonly referred to as “maternity leave”? (*n* = 100)	
No	51 (51)
Yes	49 (49)
Where can someone find information on the parental leave policies? Please select all that apply.	
Private intranet	31 (31)
Available on request	19 (19)
Included in fellowship recruitment materials	14 (14)
Provided at fellowship interviews	12 (12)
Included in employment contracts	10 (10)
Publicly accessible online	7 (7)
I am not sure	5 (5)
Other	4 (4)
To your knowledge, are fellows who take parental leave required to make up missed call shifts? (*n* = 99)	
No	84 (85)
Yes	15 (15)
To your knowledge, does your fellowship program have a written policy (separate from the ABA's or federal/state law) regarding nonchildbearing or adoptive parental leave, commonly referred to as “paternity leave” or “partner” leave? (*n* = 100)	
No	64 (64)
Yes	36 (36)
Have you had a fellow take maternity leave in the last 3 years? (*n* = 99)	
No	58 (59)
Yes	41 (41)
Have you had a fellow take paternity or partner leave in the last 3 years? (*n* = 99)	
No	73 (74)
Yes	26 (26)
Based on your observations, what is the average length of maternity leave taken by fellows in your program? (*n* = 85), weeks	
≤2	5 (6)
3–4	17 (20)
5–6	16 (19)
7–8	27 (32)
9–12	18 (21)
≥13	2 (2)
Based on your observations, what is the average length of paternity or partner leave taken by fellows in your program? (*n* = 95), weeks	
No paternity leave taken	47 (49)
≤1	16 (17)
2	19 (20)
3–5	9 (9)
6–9	3 (3)
≥10	1 (1)

^a^ When not all responders provided answers to a question, the number of surveys with complete information for that question is presented.

^b^ Values are *n* (%) unless otherwise specified.

ABA, American Board of Anesthesiology; OB, obstetrics and gynecology.

Most programs (85%) did not require fellows to make up call missed during parental leave. Sixty-six percent of programs (*N* = 63) reported paternity or partner leave of 1 week or less. Forty-nine percent of fellowship programs (*N* = 49) had a written policy about maternity leave, and 36% had a written paternity leave or partner leave policy.

Results were also analyzed with consideration of the sex of the fellowship director (Table 2). Male directors were more likely than female directors to consider it a disadvantage for an applicant to be off-cycle (*p* = 0.02). Male fellowship directors more often indicated that they believed parental leave delays subspecialty certification (*p* = 0.03). Responses were also subdivided by program subspecialty and can be found in Table 3.

**Table 2.** Demographic Characteristics of Program Directors, Policy Agreement, and Awareness According to Sex

Survey question^a^	Respondents, ***n*** (%)			***p*** ^b^
	Female (***n*** = 43)	Male (***n*** = 44)	Total (***N*** = 87)	
What is your age in years? (*n* = 42/44)				0.10^c^
30–39	17 (40)	14 (32)	31 (36)	
40–49	18 (43)	17 (39)	35 (41)	
50–59	7 (17)	10 (23)	17 (20)	
60–69	0 (0)	2 (5)	2 (2)	
≥70	0 (0)	1 (2)	1 (1)	
How many years have you been in fellowship leadership?				0.60^c^
<3	10 (23)	10 (23)	20 (23)	
3–5	13 (30)	13 (30)	26 (30)	
6–9	16 (37)	13 (30)	29 (33)	
10	4 (9)	8 (18)	12 (14)	
Do you have children?				0.31^d^
Yes	38 (88)	36 (82)	74 (85)	
No	5 (12)	8 (18)	13 (15)	
Did you (or your partner) deliver or adopt a child during your residency training?				0.53^d^
Yes	17 (40)	15 (34)	32 (37)	
No	26 (60)	29 (66)	55 (63)	
Did you (or your partner) deliver or adopt a child during your fellowship training?				0.44^d^
Not applicable, I did not have a formal fellowship	3 (7)	2 (5)	5 (6)	
No	33 (77)	31 (70)	64 (74)	
Yes	7 (16)	11 (25)	18 (21)	
How do you perceive becoming a parent impacts most anesthesiology fellows' well-being during training? (*n* = 41/44)				0.18^c^
Strong negative	1 (2)	2 (5)	3 (4)	
Some negative	6 (15)	7 (16)	13 (15)	
Slight negative	9 (22)	11 (25)	20 (24)	
None	4 (10)	7 (16)	11 (13)	
Slight positive	3 (7)	6 (14)	9 (11)	
Some positive	11 (27)	5 (11)	16 (19)	
Strong positive	7 (17)	6 (14)	13 (15)	
Parental leave delays board certification for fellows. (*n* = 43/43)				0.05^c^
Strongly disagree	2 (5)	2 (5)	4 (5)	
Disagree	9 (21)	3 (7)	12 (14)	
Somewhat disagree	3 (7)	0 (0)	3 (3)	
Neither agree nor disagree	15 (35)	16 (37)	31 (36)	
Somewhat agree	6 (14)	17 (40)	23 (27)	
Agree	6 (14)	4 (9)	10 (12)	
Strongly agree	2 (5)	1 (2)	3 (3)	
Parental leave affects job opportunities for fellows. (*n* = 43/43)				0.48^c^
Strongly disagree	3 (7)	4 (9)	7 (8)	
Disagree	10 (23)	5 (12)	15 (17)	
Somewhat disagree	6 (14)	4 (9)	10 (12)	
Neither agree nor disagree	16 (37)	23 (53)	39 (45)	
Somewhat agree	3 (7)	6 (14)	9 (10)	
Agree	3 (7)	1 (2)	4 (5)	
Strongly agree	2 (5)	0 (0)	2 (2)	
Parental leave delays subspecialty certification. (*n* = 43/43)				0.03^c^
Strongly disagree	3 (7)	2 (5)	5 (6)	
Disagree	8 (19)	2 (5)	10 (12)	
Somewhat disagree	4 (9)	1 (2)	5 (6)	
Neither agree nor disagree	15 (35)	18 (42)	33 (38)	
Somewhat agree	6 (14)	14 (33)	20 (23)	
Agree	5 (12)	6 (14)	11 (13)	
Strongly agree	2 (5)	0 (0)	2 (2)	
When reviewing applications for your fellowship program, can you identify if a resident is off-cycle?				0.46^d^
No	12 (28)	15 (34)	27 (31)	
Yes	31 (72)	29 (66)	60 (69)	
Does your program allow off-cycle residents?				0.64^d^
No	5 (12)	4 (9)	9 (10)	
Yes	38 (88)	40 (91)	78 (90)	
All else being equal, please indicate your opinion of residents being off-cycle when considering them for your fellowship program. (*n* = 38/40)				0.02^c^
Disadvantage	0 (0)	2 (5)	2 (3)	
Slight disadvantage	6 (16)	9 (22)	15 (19)	
Neither advantage nor disadvantage	27 (71)	28 (70)	55 (71)	
Slight advantage	3 (8)	1 (2)	4 (5)	
Advantage	2 (5)	0 (0)	2 (3)	
All else being equal, when considering extending interviews to residents, what is your opinion about interviewing off-cycle residents? (*n* = 38/40)				0.65^c^
Less likely to interview	0 (0)	1 (2)	1 (1)	
Slightly less likely to interview	6 (16)	2 (5)	8 (10)	
Neutral	30 (79)	37 (92)	67 (86)	
Slightly more likely to interview	1 (3)	0 (0)	1 (1)	
More likely to interview	1 (3)	0 (0)	1 (1)	
All else being equal, please indicate your opinion when ranking off-cycle residents for consideration for your fellowship program. (*n* = 38/40)				0.93^c^
Disadvantage	0 (0)	1 (2)	1 (1)	
Slight disadvantage	6 (16)	4 (10)	10 (13)	
Neither advantage nor disadvantage	31 (82)	35 (88)	66 (85)	
Slight advantage	1 (3)	0 (0)	1 (1)	
Advantage	0 (0)	0 (0)	0 (0)	

Question responses summarized by sex of program director. Data from surveys without specified sex were excluded from this summary (*n* = 14).

^a^ When not all responders provided answers to a given question, the number of surveys with complete information for that question is presented as number female/number male.

^b^ Finite population corrections are applied to all statistical tests.

^c^ Design-based Kruskal–Wallis test.

^d^ Rao–Scott chi-squared test.

**Table 3.** Summary of Program Director Responses Regarding Off-Cycle Applicants According to Program Type

Survey answer choices for each question^c^	Program type,^a,b^ ***n*** (%) (***n*** = 100)						Total
	Cardiac (***n*** = 11)	Critical care (***n*** = 18)	OB (***n*** = 23)	Pain (***n*** = 13)	Pediatric (***n*** = 29)	Regional and acute pain (***n*** = 6)	
When reviewing applications for your fellowship program, can you identify if a resident is off-cycle? (*n* = 93)							
No	5 (45)	6 (35)	5 (25)	8 (67)	5 (18)	0 (0)	29 (31)
Yes	6 (55)	11 (65)	15 (75)	4 (33)	23 (82)	5 (100)	64 (69)
Does your program allow off-cycle residents?							
No	1 (9)	1 (6)	4 (20)	2 (17)	2 (7)	0 (0)	10 (11)
Yes	10 (91)	16 (94)	16 (80)	10 (83)	26 (93)	5 (100)	83 (89)
All else being equal, please indicate your opinion of residents being off-cycle when considering them for your fellowship program. (*n* = 83)							
Disadvantage	0 (0)	0 (0)	0 (0)	1 (10)	1 (4)	0 (0)	2 (2)
Slight disadvantage	1 (10)	5 (31)	1 (6)	3 (30)	3 (12)	2 (40)	15 (18)
Neither advantage nor disadvantage	9 (90)	10 (62)	12 (75)	6 (60)	20 (77)	3 (60)	60 (72)
Slight advantage	0 (0)	1 (6)	2 (12)	0 (0)	1 (4)	0 (0)	4 (5)
Advantage	0 (0)	0 (0)	1 (6)	0 (0)	1 (4)	0 (0)	2 (2)
All else being equal, when considering extending interviews to residents, what is your opinion about interviewing off-cycle residents? (*n* = 83)							
Less likely to interview	0 (0)	0 (0)	0 (0)	0 (0)	1 (4)	0 (0)	1 (1)
Slightly less likely to interview	1 (10)	1 (6)	1 (6)	1 (10)	3 (12)	1 (20)	8 (10)
Neutral	9 (90)	15 (94)	13 (81)	9 (90)	22 (85)	4 (80)	72 (87)
Slightly more likely to interview	0 (0)	0 (0)	1 (6)	0 (0)	0 (0)	0 (0)	1 (1)
More likely to interview	0 (0)	0 (0)	1 (6)	0 (0)	0 (0)	0 (0)	1 (1)
All else being equal, please indicate your opinion when ranking off-cycle residents for consideration for your fellowship program. (*n* = 83)							
Disadvantage	0 (0)	0 (0)	0 (0)	0 (0)	1 (4)	0 (0)	1 (1)
Slight disadvantage	1 (10)	1 (6)	1 (6)	2 (20)	3 (12)	2 (40)	10 (12)
Neither advantage or disadvantage	9 (90)	15 (94)	14 (88)	8 (80)	22 (85)	3 (60)	71 (86)
Slight advantage	0 (0)	0 (0)	1 (6)	0 (0)	0 (0)	0 (0)	1 (1)
Advantage	0 (0)	0 (0)	0 (0)	0 (0)	0 (0)	0 (0)	0 (0)

^a^ Data from surveys without specified program type were excluded from this summary (*n* = 1).

^b^ Finite population corrections are applied to all statistical tests.

^c^ When not all responders provided answers to a given question, the number of surveys with complete information for that question is presented.

**FIG. 1.**
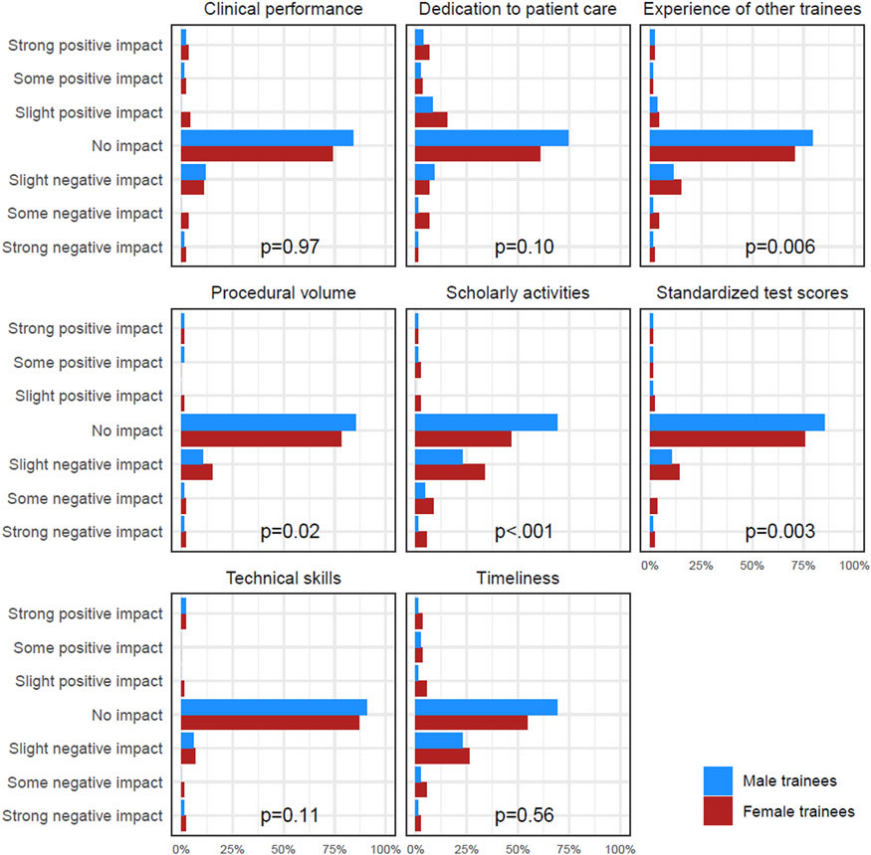
Results of a nationwide survey of fellowship directors' perceptions of the impact of fellows becoming a parent during their training by sex of the trainee. The survey was conducted in January 2019.

Male and female fellowship directors reported their perception of the impact that becoming a parent had on the fellow's training (Fig. 1). A slight negative effect was perceived on scholarly activities, standardized test scores, and timeliness for male and female trainees who became parents during their fellowship. However, program directors perceived a greater negative impact for female trainees on scholarly activities, procedural volume, and standardized test scores. Of interest, program directors perceived a positive effect of parenthood on dedication to patient care in female and male trainees.

### Off-cycle resident applicants and fellows

The majority (69%) of fellowship directors reported that they can tell when a resident is off-cycle from the application. Of program directors, 11% (*n* = 10/93) reported that they do not allow off-cycle residents in their program. In the consideration, interview, and rank of off-cycle applicants, the responses varied with subspecialty type, but that variation did not reach statistical significance (*p* = 0.16, 0.97, and 0.51, respectively). When delineated by specialty, 20% (*n* = 4/20) of the obstetrics and gynecology (OB) fellowship program directors and 17% (*n* = 2/12) of the pain program directors reported that they do not allow off-cycle applicants. There was not a statistically significant difference among program types with respect to allowing off-cycle applicants (*p* = 0.39).

Program directors who confirmed acceptance of off-cycle applicants were asked further questions. When considering off-cycle applicants and with all qualifications being equal, 71% of fellowship directors (*n* = 60) stated it is neither an advantage nor a disadvantage; 8% stated it as either a slight advantage or an advantage; and 20% stated it as a slight disadvantage or a disadvantage. No significant differences were observed among specialties for this survey aspect (*p* = 0.16). When considering extending interviews to off-cycle candidates, 86% of respondents felt neutral (*n* = 72), 4% (*n* = 3) were slightly more likely or more likely, and 11% were slightly less likely or less likely.

In considering ranking off-cycle candidates, 85% (*n* = 71) stated it is neither an advantage nor a disadvantage; 1% stated it a slight advantage; and 14% stated it a slight disadvantage or a disadvantage. The detailed responses by subspecialty are included in Supplementary Table S1.

Asked about the advantages of having off-cycle residents in a fellowship program, survey participants chose the following (and were encouraged to choose all that applied): train new fellows (*n* = 52 [51%]), cover additional call shifts while new fellows train (*n* = 21 [21%]), recruitment (*n* = 15 [15%]), orientation is off-cycle (*n* = 9 [9%]), clinical assignments (*n* = 7 [7%]), and curriculum planning (*n* = 6 [6%]). Twenty-four directors (24%) cited no advantages.

When asked about the disadvantages of off-cycle residents joining a fellowship program, respondents expressed the following: orientation is off-cycle (*n* = 54 [53%]), board certification is delayed (*n* = 33 [33%]), curriculum planning (*n* = 25 [25%]), scheduling is difficult (*n* = 24 [24%]), evaluation and assessments (*n* = 14 [14%]), postgraduate placements (*n* = 7 [7%]), and recruitment (*n* = 5 [5%]). Twenty directors (20%) cited no disadvantages.

The survey asked how a program schedules the fellows who extend their graduation beyond July 1, as this is the typical start time. Some fellows are scheduled on standard rotations (*n* = 70 [69%]) or on the rotations they missed during parental leave (*n* = 49 [49%]). Some fellows train new fellows (*n* = 25 [25%]), use elective or research time (*n* = 21 [21%]), cover additional call shifts while new fellows are trained (*n* = 12 [12%]) and are scheduled based on staffing needs (*n* = 4 [4%]).

## Discussion

We examined the perceptions of anesthesiology fellowship program directors about the impact of parental leave on the fellow's training, well-being, and performance during fellowship and also evaluated the directors' impression of off-cycle fellowship applicants.

Little data have been reported about fellowship directors' perceptions. However, there is increasing interest in the literature about the perceptions of residency program directors about parental leave among various surgical specialties.^6,8,9^ Sharpe et al.^7^ evaluated the perceptions of 56 anesthesiology residency program directors on parental leave; when asked how parental leave affects residents' futures, 24/50 (48%) program directors felt it delayed board certification and 28/50 (56%) thought it affected fellowship opportunities. When compared with male trainees, program directors perceived that becoming a parent negatively affected female trainees' timeliness, technical skills, scholarly activities, procedural volume, and standardized test scores.

We found similar perceptions of anesthesiology fellowship program directors in our current study with a perceived greater negative impact on scholarly activities, procedural volume, standardized test scores, and affects training experience of other residents for female trainees than male trainees. These perceptions are pervasive in anesthesiology and similar to surgery, OB, and ophthalmology.

In a study of 644 trainees from all medical specialties, 38% of women and 20% of men reported delaying pregnancy until after training.^2^ Women residents were more likely to cite concerns about extension of training, interference with fellowship plans, and potential for pregnancy complications. In a pilot survey, 37 women anesthesiologists gave birth during their training, 56% extended their training, 10% delayed board certification, and 38% believed that they were at a disadvantage in job or fellowship applications.^10^ A study of 1,827 women anesthesiologists found that 35% altered the desired number of children or their desired age of childbearing because of work demands.^11^

The present survey showed greater negative perceptions about off-cycle fellowship applicants than the investigators anticipated. Most program directors responded that based on the application, they can identify whether an applicant is off-cycle. Among the fellowship directors, 11%, including almost 20% of directors of obstetric anesthesia and pain fellowships, do not allow off-cycle applicants in their program, regardless of an applicant's qualifications or academic performance.

Of the programs that allow off-cycle applicants, 20% of fellowship directors believed that for an applicant, being off-cycle was a disadvantage, and these directors were less likely to interview or rank such candidates, further exemplifying a bias against this subset of residents. This perception diminishes the number of fellowship positions open to off-cycle residents.

There are multiple causes for residents graduating off-cycle and could include other health reasons rather than parental leave. While we did not delineate in the survey the different causes of residents graduating off-cycle, it stands to reason that a significant proportion of this group is off-cycle due to parental leave. This is an important finding because it could have substantial long-term implications on an individual trainee. For instance, an individual may have wanted to pursue a particular fellowship, such as becoming a cardiac anesthesiologist or pain physician, but may not if programs do not allow off-cycle residents. The trainee may decide to delay the fellowship and enter the job market or they may abandon their goal of that additional training all together.

Fellowship directors may be reluctant to include off-cycle residents because they may present scheduling and orientation challenges. Other specialties have recognized problems with the July 1 timing of transition from residency to fellowship and have adopted delayed fellowship orientation and start dates. Orthopedic surgery fellowships agreed on an August 1 start in the 1990s, and in 2014, the American Board of Surgery^12^ moved all fellowships to a uniform start date of August 1. Delayed starts allow residents to complete all clinical requirements without taking vacation or other methods to move and begin fellowship orientation on time.^13^

A survey of the Association of Program Directors in Radiology indicated that (1) fellowship orientations before July 1 cause residency staffing problems and (2) 78% of program directors supported a delay to the start of radiology fellowships.^14^ In 2020, the Association of Medical School Pediatric Department Chairs (AMSPDC) released survey data showing that 94% of fellows and fellowship directors in delayed-start programs were satisfied with the delay.^15^ Of programs without a delayed start date, 92% of fellows would have preferred their programs to follow the AMSPDC recommendations. Universal delayed start dates for anesthesiology fellowships may resolve this possible bias against off-cycle applicants that was observed in this study.

Policies that exclude applicants because of the fellowship start date can put the affected applicants at a disadvantage and potentially exclude them from fellowship opportunities altogether. Women may perceive these potential disadvantages (*e.g.*, limited off-cycle opportunities) and alter their childbearing plans accordingly, which may have detrimental effects on their satisfaction with the specialty. In a pilot survey, colleagues or staff discouraged 26.8% of women anesthesiologists from becoming pregnant or breastfeeding, and less than one-half of the anesthesiologist respondents who gave birth during residency or fellowship reported adequate parental leave, lactation facilities, or lactation duration.^10^ Of those who delayed training, 38% felt at a disadvantage when applying for a job or fellowship.

Anesthesiology has the highest rate of maternal discrimination among all major specialties.^16^ Factors contributing to this finding for women anesthesiologists have not been fully explored. A recent study reported that a strong association was found between women who altered their age of childbearing or the number of children because of work demands and ultimately a recommendation against anesthesiology (risk ratio, 5.1).^11^

A follow-up survey of anesthesiology fellows who have completed residency training after 2019 will be important to assess the effects of the ABA extended leave of absence policy on the training, employment, and board certification rate.

### Limitations

The present study has several limitations, many of which are present with any survey. We selected a survey instrument that had been used previously in anesthesiology residency programs, with the hypothesis that anesthesiology fellows face similar challenges in family and work life. The survey response rate of 31% may reflect a nonresponse bias. Program directors self-reported this information and may recall incorrectly some of the information, leading to recall bias. Because we were able to send e-mails through the Society of Pediatric Anesthesiology and the Society of Obstetric Anesthesia and Perinatology Listservs, we had higher response rates for those specialties.

## Conclusion

Anesthesiology fellowship program directors perceive a more negative impact from women having children during fellowship compared with men. Also, the findings regarding off-cycle resident applicants for fellowship could have a negative impact on fellowship opportunities for the residents. These differences may have important implications throughout the career of men and women anesthesiologists.

## Supplementary Material

Supplemental data

Supplemental data

## Abbreviations Used

ABA: American Board of Anesthesiology
ACGME: Accreditation Council for Graduate Medical Education
AMSPDC: Association of Medical School Pediatric Department Chairs
FPC: finite population correction
OB: obstetrics and gynecology
