# Supplementary material for: A Nationwide Cross-Sectional Survey of Anesthesiology Fellowship Program Directors: Attitudes on Parental Leave in Residency and Fellowship Training
Source: Womens Health Rep (New Rochelle). 2022 May 6;3(1):395–404. doi: 10.1089/whr.2021.0130 (PMC9148645; doi:10.1089/whr.2021.0130)
Supplement: Supplemental data [file Suppl_Data.docx]

Supplementary Data

Fellowship Directors Survey

Start of Block: Default Block

Q43 Are you a fellowship director?

- Yes (1)
- No (2)

Q44 What type of anesthesiology fellowship do you direct?

- Cardiac (1)
- Pediatric (2)
- OB (3)
- Pain (4)
- Regional and Acute Pain (5)
- Critical Care (6)
- Neuroanesthesia (7)
- Practice Management (8)
- Public Policy (9)
- Transplant (10)
- Trauma (11)
- Other, please specify (12) ________________________________________________

Q45 Is your fellowship accredited?

- Yes (1)
- No (2)

Q2 What type of facility is your fellowship program a part of?

- Community (1)
- Military (2)
- Academic (3)
- Other, please specify (4) ________________________________________________

Q3 What region is your fellowship program located in?

- East North Central (IL,IN,MI,OH,WI) (1)
- East South Central (AL,KY,MS,TN) (2)
- Mid Atlantic (NJ,NY,PA) (3)
- Mountain (AZ,CO,ID,MT,NM,NV,UT,WY) (4)
- New England (CT,MA,ME,NH,RI,VT) (5)
- Pacific (AK,CA,HI,OR,WA) (6)
- South Atlantic (DC,DE,FL,GA,MD,NC,SC,VA,WV) (7)
- Territory (PR) (8)
- West North Central (IA,KS,MN,MO,ND,NE,SD) (9)
- West South Central (AR,LA,OK,TX) (10)

Q4 How many total fellows do you have in your program?

________________________________________________________________

Q5 Percentage of female fellows in your program (as a percentage of total fellows in the fellowship program):

- 0-20% (1)
- 21-40% (2)
- 41-60% (3)
- 61-80% (4)
- 81-100% (5)

Q6 Percentage of fellows (male and female) with children:

- 0-20% (1)
- 21-40% (2)
- 41-60% (3)
- 61-80% (4)
- 81-100% (5)

Q10 To your knowledge, does your fellowship program have a written policy (separate from the ABA’s or federal/state law) regarding childbearing parental leave, commonly referred to as “maternity leave”?

- Yes (1)
- No (2)

Q11 To your knowledge, does your fellowship program have a written policy (separate from the ABA’s or federal/state law) regarding non-childbearing or adoptive parental leave, commonly referred to as “paternity leave” or “partner” leave?

- Yes (1)
- No (2)

Display This Question:

If To your knowledge, does your fellowship program have a written policy (separate from the ABA’s or... = Yes

Or To your knowledge, does your fellowship program have a written policy (separate from the ABA’s or... = Yes

Q12 Where can someone find information on the parental leave policies? Please select all that apply.

- a. Publicly accessible online (1)
- b. Private intranet (2)
- c. Included in employment contracts (3)
- d. Included in fellowship recruitment materials (4)
- e. Provided at fellowship interviews (5)
- f. Available upon request (6)
- g. I am not sure (7)
- h. Other, please specify (8) ________________________________________________

End of Block: Default Block

Start of Block: Fellowship Parental Leave - Program Experience

Q13 Have you had a fellow take **maternity leave** in the last 3 years?

- Yes (1)
- No (2)

Display This Question:

If Have you had a fellow take maternity leave in the last 3 years? = Yes

Q14 How many fellows have taken **maternity leave** in the past 3 years?

________________________________________________________________

Q15 Based on your observations, what is the average length of **maternity leave** taken by fellows in your program?

- a. Less than or equal to 2 weeks (1)
- b. 3-4 weeks (2)
- c. 5-6 weeks (3)
- d. 7-8 weeks (4)
- e. 9-12 weeks (5)
- f. 13 weeks or more (6)

| Page Break |  |
| --- | --- |

Q42 Have you had a fellow take **paternity or partner leave** in the last 3 years?

- Yes (1)
- No (2)

Display This Question:

If Have you had a fellow take paternity or partner leave in the last 3 years? = Yes

Q40 How many fellows have taken **paternity or partner leave** in the past 3 years?

________________________________________________________________

Q16 Based on your observations, what is the average length of **paternity or partner leave** taken by fellows in your program

- a. No paternity leave taken (1)
- b. Less than or equal to 1 weeks (2)
- c. 2 weeks (3)
- d. 3-5 weeks (4)
- e. 6-9 weeks (5)
- f. 10 weeks or more (6)

| Page Break |  |
| --- | --- |

Q17 Who covers clinical duties for fellows in your program who take parental leave? Select all that apply.

- a. Residents (1)
- b. Fellows (2)
- c. Attendings (3)
- d. Employed CRNAs or AAs (4)
- e. Moonlighters (MD or CRNA), hired coverage (5)
- f. Coverage typically not necessary (6)
- g. Other, please specify (7) ________________________________________________

Display This Question:

If Who covers clinical duties for fellows in your program who take parental leave? Select all that a... = a. Residents

Or Who covers clinical duties for fellows in your program who take parental leave? Select all that a... = b. Fellows

Q18 To your knowledge, do other residents or fellows receive additional financial compensation for covering for fellows on parental leave?

- Yes (1)
- No (2)

Display This Question:

If Who covers clinical duties for fellows in your program who take parental leave? Select all that a... = a. Residents

Or Who covers clinical duties for fellows in your program who take parental leave? Select all that a... = b. Fellows

Q19 To your knowledge, are other fellows or residents compensated with special schedule arrangements for covering for fellows on parental leave? Special schedule arrangements can include shorter shifts, additional time off in the future, etc.

- Yes (1)
- No (2)

Q21 Can call shifts go uncovered by fellows in your fellowship program?

- Yes (1)
- No (2)

Display This Question:

If Can a rotation go uncovered by fellows in your fellowship program? = Yes

Or Can call shifts go uncovered by fellows in your fellowship program? = Yes

Q22 What amount of rotations or calls can go uncovered by fellows in your fellowship program?

- None at all – all rotations and calls must be covered by fellows (1)
- Few rotations/calls (2)
- Some rotations/calls (3)
- Most rotations/calls (4)
- All rotations/calls (5)

Q23  To your knowledge, are fellows who take parental leave required to make up missed call shifts?

- Yes (1)
- No (2)

Display This Question:

If  To your knowledge, are fellows who take parental leave required to make up missed call shifts? = Yes

Q24 When do fellows make up missed call shifts? Select all that apply.

- a. Take additional call shifts in advance of parental leave. (1)
- b. Take additional call shifts upon return to work from leave. (2)
- c. Take additional call shifts during extension of training (i.e. July/August after typical fellow year). (3)
- d. Other (please specify): (4) ________________________________________________

Q25 To your knowledge, what type of time is used to cover a parental leave in your program? Select all that apply.

- a. Sick time (1)
- b. Vacation time (2)
- c. Disability (3)
- d.Unpaid time off (4)

Q26 How does your program typically schedule fellows who extend their graduation beyond July 1st?  Select all that apply.

- a. Fellows are scheduled on standard rotations during the extension time. (1)
- b. Fellows are utilized for training new fellows during the extension time. (2)
- c. Fellows cover additional call shifts while new fellows are trained. (3)
- d. Fellows are scheduled on the rotations in the extension time that were missed during parental leave. (4)
- e. Fellows are scheduled on rotations during the extension time based on staffing needs, even if the fellow has already completed the required rotation. (5)
- f. Fellows can take elective, research, or nonclinical rotations during the extension time. (6)
- h. Other, please specify (7) ________________________________________________

End of Block: Fellowship Parental Leave - Program Experience

Start of Block: Questions Regarding Fellows with Children

Q27 How do you perceive becoming a parent impacts most anesthesiology fellows' well-being during training?

- a. Strong negative impact (1)
- b. Some negative impact (2)
- c. Slight negative impact (3)
- d. No impact (4)
- e. Slight positive impact (5)
- f. Some positive impact (6)
- g. Strong positive impact (7)

Q28 Consider **female** trainees who become mothers during fellowship.  How does becoming a parent impact the following aspects of a female trainee’s work?

|  | Strong negative impact (1) | Some negative impact (2) | Slight negative impact (3) | No impact (4) | Slight positive impact (5) | Some positive impact (6) | Strong positive impact (7) |
| --- | --- | --- | --- | --- | --- | --- | --- |
| Timeliness (1) |  |  |  |  |  |  |  |
| Dedication to patient care (2) |  |  |  |  |  |  |  |
| Clinical performance (3) |  |  |  |  |  |  |  |
| Technical skills (4) |  |  |  |  |  |  |  |
| Scholarly activities (5) |  |  |  |  |  |  |  |
| Procedural volume (6) |  |  |  |  |  |  |  |
| Standardized test scores (7) |  |  |  |  |  |  |  |
| Affects training experience of other fellows (8) |  |  |  |  |  |  |  |

| Page Break |  |
| --- | --- |

Q30 Consider **male** trainees who become fathers during fellowship.  How does becoming a parent impact the following aspects of a male trainee’s work?

|  | Strong negative impact (1) | Some negative impact (2) | Slight negative impact (3) | No impact (4) | Slight positive impact (5) | Some positive impact (6) | Strong positive impact (7) |
| --- | --- | --- | --- | --- | --- | --- | --- |
| Timeliness (1) |  |  |  |  |  |  |  |
| Dedication to patient care (2) |  |  |  |  |  |  |  |
| Clinical performance (3) |  |  |  |  |  |  |  |
| Technical skills (4) |  |  |  |  |  |  |  |
| Scholarly activities (5) |  |  |  |  |  |  |  |
| Procedural volume (6) |  |  |  |  |  |  |  |
| Standardized test scores (e.g., ITE, AKT, etc.) (7) |  |  |  |  |  |  |  |
| Affects training experience of other fellows (8) |  |  |  |  |  |  |  |

Q29  Please describe any other impact that becoming a parent has on work and performance of fellows that has not been covered in the questions above.

________________________________________________________________

End of Block: Questions Regarding Fellows with Children

Start of Block: Attitude regarding off-cycle residents for consideration for fellowship

Q51 When reviewing applications for your fellowship program, can you identify if a resident is "off-cycle?" (Their expected completion of residency is different than their peer group)

- Yes (1)
- No (2)

Q52 Does your program allow "off-cycle" residents?

- Yes (1)
- No (2)

Display This Question:

If Does your program allow "off-cycle" residents? = Yes

Q46 All else being equal, please indicate your opinion of a resident being "off-cycle" when considering them for your fellowship program.

- Advantage (1)
- Slight advantage (2)
- Neither advantage or disadvantage (3)
- Slight disadvantage (4)
- Disadvantage (5)

Display This Question:

If Does your program allow "off-cycle" residents? = Yes

Q47 All else being equal, when considering extending interviews to residents, what is your opinion about interviewing “off-cycle” residents?

- More likely to interview (1)
- Slightly more likely to interview (2)
- Neutral (3)
- Slightly less likely to interview (4)
- Less likely to interview (5)

Display This Question:

If Does your program allow "off-cycle" residents? = Yes

Q48 All else being equal, please indicate your opinion when ranking "off-cycle" residents for consideration for your fellowship program.

- Advantage (1)
- Slight advantage (2)
- Neither advantage or disadvantage (3)
- Slight disadvantage (4)
- Disadvantage (5)

Q49 What are the advantages of having "off-cycle" residents join your program? Select all that apply.

- Train new fellows (1)
- Cover additional call shifts while new fellows train (2)
- Orientation is off-cycle (3)
- Board certification is delayed (4)
- Recruitment (5)
- Curriculum planning (6)
- Clinical assignments (7)
- Post graduate placements (8)
- Evaluation and Assessments (9)
- No advantages (10)
- Other (11) ________________________________________________

Q50 What are the disadvantages of having off-cycle residents join your program? Select all that apply.

- Orientation is off-cycle (1)
- Scheduling is difficult (2)
- Board certification is delayed (3)
- Recruitment (4)
- Curriculum planning (5)
- Clinical assignments (6)
- Post-graduate placements (7)
- Evaluation and assessments (8)
- No disadvantages (9)
- Other (10) ________________________________________________

End of Block: Attitude regarding off-cycle residents for consideration for fellowship

Start of Block: Attitude on Accreditation Policies

Q31 Do you agree or disagree with the following

|  | Strongly disagree (1) | Disagree (2) | Somewhat disagree (3) | Neither agree nor disagree (4) | Somewhat agree (5) | Agree (6) | Strongly agree (7) |
| --- | --- | --- | --- | --- | --- | --- | --- |
| Parental leave delays board certification for fellows (1) |  |  |  |  |  |  |  |
| Parental leave affects job opportunities for fellows (2) |  |  |  |  |  |  |  |
| Parental leave delays subspecialty certification (3) |  |  |  |  |  |  |  |

Q32 Please describe any other comments you have about parental leave for anesthesiology fellows that have not been covered in the questions above

________________________________________________________________

End of Block: Attitude on Accreditation Policies

Start of Block: Demographics

Q33 What is your sex?

- Male (1)
- Female (2)
- Another gender identity (3)
- I prefer not to identify (4)

Q34 What is your age (in years):

- a. 30-39 (1)
- b.40-49 (2)
- c. 50-59 (3)
- d. 60-69 (4)
- e.≥70 Choice 5 (5)

Q35 How many years have you been in fellowship leadership?

- a. < 3 years (1)
- b. 3-5 years (2)
- c. 6-9 years (3)
- d. ≥10 years (4)

Q36 Do you have children?

- a. Yes (1)
- b. No (2)

Q37 Did you (or your partner) deliver or adopt a child during your **residency** training?

- a. Yes (1)
- b. No (2)

Q38 Did you (or your partner) deliver or adopt a child during your **fellowship** training?

- Yes (1)
- No (2)
- NA, I did not have a formal fellowship (3)

End of Block: Demographics
