# Supplementary material for: A Nationwide Cross-Sectional Survey of Anesthesiology Fellowship Program Directors: Attitudes on Parental Leave in Residency and Fellowship Training
Source: Womens Health Rep (New Rochelle). 2022 May 6;3(1):395–404. doi: 10.1089/whr.2021.0130 (PMC9148645; doi:10.1089/whr.2021.0130)
Supplement: Supplemental data [file Suppl_TableS1.docx]

Supplementary Table S1**.** Summary of Program Director Responses According to Program Type

|  | **Program type,^a,b^ Number (%) (n=100)** | | | | | |  |
| --- | --- | --- | --- | --- | --- | --- | --- |
| **Survey answer choices for each question^c^** | **Cardiac (n=11)** | **Critical care (n=18)** | **OB (n=23)** | **Pain (n=13)** | **Pediatric (n=29)** | **Regional and acute pain (n=6)** | **Total** |
| What type of facility is your fellowship program a part of? | | | | | | |  |
| Academic | 11 (100) | 18 (100) | 22 (96) | 11 (85) | 28 (97) | 6 (100) | 96 (96) |
| Community | 0 (0) | 0 (0) | 1 (4) | 0 (0) | 0 (0) | 0 (0) | 1 (1) |
| Military | 0 (0) | 0 (0) | 0 (0) | 2 (15) | 0 (0) | 0 (0) | 2 (2) |
| Other (please specify) | 0 (0) | 0 (0) | 0 (0) | 0 (0) | 1 (3) | 0 (0) | 1 (1) |
| Region of US (n=98) |  |  |  |  |  |  |  |
| East | 5 (45) | 7 (39) | 4 (19) | 2 (15) | 5 (17) | 2 (33) | 25 (26) |
| Midwest | 1 (9) | 5 (28) | 7 (33) | 2 (15) | 12 (41) | 1 (17) | 28 (29) |
| West | 2 (18) | 2 (11) | 3 (14) | 3 (23) | 3 (10) | 1 (17) | 14 (14) |
| Southeast | 1 (9) | 1 (6) | 3 (14) | 2 (15) | 3 (10) | 2 (33) | 12 (12) |
| South | 2 (18) | 3 (17) | 4 (19) | 4 (31) | 6 (21) | 0 (0) | 19 (19) |
| Percentage of female fellows in your fellowship program (as percentage of total fellows in program) (n=98) | | | | | | | |
| 0-20 | 5 (45) | 8 (44) | 5 (24) | 7 (54) | 2 (7) | 1 (17) | 28 (29) |
| 21-40 | 2 (18) | 3 (17) | 2 (10) | 5 (38) | 1 (3) | 0 (0) | 13 (13) |
| 41-60 | 3 (27) | 6 (33) | 1 (5) | 1 (8) | 15 (52) | 3 (50) | 29 (30) |
| 61-80 | 0 (0) | 1 (6) | 3 (14) | 0 (0) | 5 (17) | 0 (0) | 9 (9) |
| 81-100 | 1 (9) | 0 (0) | 10 (48) | 0 (0) | 6 (21) | 2 (33) | 19 (19) |
| Percentage of fellows (male and female) with children (n=98) | | | | | | |  |
| 0-20 | 7 (64) | 5 (28) | 10 (48) | 4 (31) | 10 (34) | 5 (83) | 41 (42) |
| 21-40 | 2 (18) | 3 (17) | 2 (10) | 0 (0) | 4 (14) | 0 (0) | 11 (11) |
| 41-60 | 2 (18) | 2 (11) | 2 (10) | 5 (38) | 8 (28) | 1 (17) | 20 (20) |
| 61-80 | 0 (0) | 5 (28) | 3 (14) | 2 (15) | 3 (10) | 0 (0) | 13 (13) |
| 81-100 | 0 (0) | 3 (17) | 4 (19) | 2 (15) | 4 (14) | 0 (0) | 13 (13) |
| To your knowledge, does your fellowship program have a written policy (separate from the ABA’s or federal/state law) regarding childbearing parental leave, commonly referred to as *maternity leave*? | | | | | | | |
| No | 5 (45) | 8 (44) | 14 (61) | 7 (54) | 14 (48) | 3 (50) | 51 (51) |
| Yes | 6 (55) | 10 (56) | 9 (39) | 6 (46) | 15 (52) | 3 (50) | 49 (49) |
| To your knowledge, does your fellowship program have a written policy (separate from the ABA’s or federal/state law) regarding nonchildbearing or adoptive parental leave, commonly referred to as *paternity leave* or *partner leave*? | | | | | | | |
| No | 5 (45) | 12 (67) | 18 (78) | 8 (62) | 18 (62) | 3 (50) | 64 (64) |
| Yes | 6 (55) | 6 (33) | 5 (22) | 5 (38) | 11 (38) | 3 (50) | 36 (36) |
| Have you had a fellow take maternity leave in the last 3 years? (n=98) | | | | | | |  |
| No | 8 (73) | 10 (56) | 16 (73) | 9 (69) | 12 (41) | 2 (40) | 57 (58) |
| Yes | 3 (27) | 8 (44) | 6 (27) | 4 (31) | 17 (59) | 3 (60) | 41 (42) |
| Based on your observations, what is the average length of maternity leave taken by fellows in your program? (n=84) | | | | | | | |
| ≤2 wk | 1 (12) | 2 (12) | 0 (0) | 2 (15) | 0 (0) | 0 (0) | 5 (6) |
| 3-4 wk | 2 (25) | 7 (41) | 1 (6) | 1 (8) | 6 (24) | 0 (0) | 17 (20) |
| 5-6 wk | 2 (25) | 3 (18) | 4 (24) | 3 (23) | 4 (16) | 0 (0) | 16 (19) |
| 7-8 wk | 2 (25) | 4 (24) | 5 (29) | 2 (15) | 10 (40) | 3 (75) | 26 (31) |
| 9-12 wk | 1 (12) | 1 (6) | 5 (29) | 5 (38) | 5 (20) | 1 (25) | 18 (21) |
| ≥13 wk | 0 (0) | 0 (0) | 2 (12) | 0 (0) | 0 (0) | 0 (0) | 2 (2) |
| Based on your observations, what is the average length of paternity or partner leave taken by fellows in your program? (n=94) | | | | | | | |
| No leave taken | 3 (27) | 11 (61) | 13 (68) | 5 (38) | 12 (43) | 3 (60) | 47 (50) |
| ≤1 wk | 4 (36) | 1 (6) | 3 (16) | 3 (23) | 4 (14) | 1 (20) | 16 (17) |
| 2 wk | 3 (27) | 5 (28) | 0 (0) | 4 (31) | 7 (25) | 0 (0) | 19 (20) |
| 3-5 wk | 0 (0) | 1 (6) | 1 (5) | 1 (8) | 4 (14) | 1 (20) | 8 (9) |
| 6-9 wk | 1 (9) | 0 (0) | 1 (5) | 0 (0) | 1 (4) | 0 (0) | 3 (3) |
| ≥10 wk | 0 (0) | 0 (0) | 1 (5) | 0 (0) | 0 (0) | 0 (0) | 1 (1) |
| Do you have children? (n=90) | | | | | | |  |
| Yes | 9 (82) | 12 (71) | 17 (85) | 10 (91) | 24 (92) | 5 (100) | 77 (86) |
| No | 2 (18) | 5 (29) | 3 (15) | 1 (9) | 2 (8) | 0 (0) | 13 (14) |
| Did you (or your partner) deliver or adopt a child during your residency training? (n=89) | | | | | | |  |
| Yes | 0 (0) | 4 (25) | 12 (60) | 5 (45) | 10 (38) | 1 (20) | 32 (36) |
| No | 11 (100) | 12 (75) | 8 (40) | 6 (55) | 16 (62) | 4 (80) | 57 (64) |
| Did you (or your partner) deliver or adopt a child during your fellowship training? (n=89) | | | | | | |  |
| Not applicable, I did not have a formal fellowship | 0 (0) | 1 (6) | 4 (20) | 0 (0) | 0 (0) | 0 (0) | 5 (6) |
| No | 11 (100) | 11 (69) | 13 (65) | 7 (64) | 19 (73) | 4 (80) | 65 (73) |
| Yes | 0 (0) | 4 (25) | 3 (15) | 4 (36) | 7 (27) | 1 (20) | 19 (21) |
| Parental leave delays board certification for fellows. (n=90) | | | | | | |  |
| Strongly disagree | 2 (18) | 1 (6) | 0 (0) | 1 (8) | 1 (4) | 0 (0) | 5 (6) |
| Disagree | 3 (27) | 0 (0) | 4 (20) | 4 (33) | 1 (4) | 1 (20) | 13 (14) |
| Somewhat disagree | 1 (9) | 0 (0) | 2 (10) | 0 (0) | 0 (0) | 0 (0) | 3 (3) |
| Neither agree nor disagree | 4 (36) | 5 (31) | 6 (30) | 3 (25) | 10 (38) | 4 (80) | 32 (36) |
| Somewhat agree | 1 (9) | 8 (50) | 4 (20) | 1 (8) | 10 (38) | 0 (0) | 24 (27) |
| Agree | 0 (0) | 2 (12) | 3 (15) | 2 (17) | 3 (12) | 0 (0) | 10 (11) |
| Strongly agree | 0 (0) | 0 (0) | 1 (5) | 1 (8) | 1 (4) | 0 (0) | 3 (3) |
| Parental leave affects job opportunities for fellows. (n=90) | | | | | | |  |
| Strongly disagree | 2 (18) | 2 (12) | 1 (5) | 2 (17) | 1 (4) | 0 (0) | 8 (9) |
| Disagree | 2 (18) | 3 (19) | 4 (20) | 3 (25) | 2 (8) | 1 (20) | 15 (17) |
| Somewhat disagree | 3 (27) | 1 (6) | 3 (15) | 1 (8) | 3 (12) | 0 (0) | 11 (12) |
| Neither agree nor disagree | 4 (36) | 7 (44) | 7 (35) | 6 (50) | 14 (54) | 3 (60) | 41 (46) |
| Somewhat agree | 0 (0) | 2 (12) | 2 (10) | 0 (0) | 4 (15) | 1 (20) | 9 (10) |
| Agree | 0 (0) | 1 (6) | 1 (5) | 0 (0) | 2 (8) | 0 (0) | 4 (4) |
| Strongly agree | 0 (0) | 0 (0) | 2 (10) | 0 (0) | 0 (0) | 0 (0) | 2 (2) |
| Parental leave delays subspecialty certification. (n=90) | | | | | | |  |
| Strongly disagree | 2 (18) | 1 (6) | 1 (5) | 1 (8) | 1 (4) | 0 (0) | 6 (7) |
| Disagree | 2 (18) | 1 (6) | 2 (10) | 3 (25) | 1 (4) | 1 (20) | 10 (11) |
| Somewhat disagree | 1 (9) | 1 (6) | 2 (10) | 0 (0) | 1 (4) | 0 (0) | 5 (6) |
| Neither agree nor disagree | 5 (45) | 3 (19) | 8 (40) | 5 (42) | 11 (42) | 3 (60) | 35 (39) |
| Somewhat agree | 1 (9) | 7 (44) | 4 (20) | 0 (0) | 8 (31) | 1 (20) | 21 (23) |
| Agree | 0 (0) | 3 (19) | 2 (10) | 2 (17) | 4 (15) | 0 (0) | 11 (12) |
| Strongly agree | 0 (0) | 0 (0) | 1 (5) | 1 (8) | 0 (0) | 0 (0) | 2 (2) |
|  |  |  |  |  |  |  |  |

Abbreviations: ABA, American Board of Anesthesiology; OB, obstetrics and gynecology.

^a^ Data from surveys without specified program type were excluded from this summary (n=1).

^b^ Finite population corrections are applied to all statistical tests.

^c^ When not all responders provided answers to a given question, the number of surveys with complete information for that question is presented.
